# Multiple-Antenna Emitters Identification Based on a Memoryless Power Amplifier Model

**DOI:** 10.3390/s19235233

**Published:** 2019-11-28

**Authors:** Jun Lu, Xiaodong Xu

**Affiliations:** Department of Electrical Engineering and Information Science, University of Science and Technology of China, Hefei 230026, China; lujun052@mail.ustc.edu.cn

**Keywords:** specific emitter identification, multiple antennas, power amplifier nonlinearity, nonlinear least square

## Abstract

Power amplifier (PA) nonlinearity is typically unique at the radio frequency (RF) front-end for particular emitters. It can play a crucial role in the application of specific emitter identification (SEI). In this paper, under the Multi-Input Multi-Output (MIMO) multipath communication scenario, two data-aided approaches are proposed to identify multi-antenna emitters using PA nonlinearity. Built upon a memoryless polynomial model, the first approach formulates a linear least square (LLS) problem and presents the closed-form solution of nonlinear coefficients in a MIMO system by means of singular value decomposition (SVD) operation. Another alternative approach estimates nonlinear coefficients of each individual PA through nonlinear least square (NLS) solved by the regularized Gauss–Newton iterative scheme. Moreover, there are some practical discussions of our proposed approaches about the mismatch of the order of PA model and the rank-deficient condition. Finally, the average misclassification rate is derived based on the minimum error probability (MEP) criterion, and the proposed approaches are validated to be effective through extensively numerical simulations.

## 1. Introduction

Specific emitter identification (SEI) is committed to distinguish individual radiation sources by using essential radio frequency fingerprint (RFF) features extracted from different emitters. It can be applied to military communication [1], physical layer authentication [2], and enhancement of the security in wireless network, such as very high frequency (VHF) radio networks, Wi-Fi networks, cognitive radios, cellular networks [3], and so on.

In general, based on different states of signals, the identifiable RFF features for SEI are usually extracted from the transient signal or the steady-state signal. The transient signal, actually the turn-on signal, carries unique and unintentional information that is advantageous to emitter identification, and the features underlying are mainly extracted from the instantaneous amplitude, phase, frequency, and energy envelope [4,5,6]. Nevertheless, it is difficult to capture the transient signal since the duration time is often too short to use. As for the steady-state signal, many researchers focus on extracting the distinguishable features that are generated by hardware imperfection of the components inside the radiation source through advanced signal processing techniques. On one hand, the statistical characteristics of the original RF signal, such as the high order spectrum, have been used as the features to identify different emitters [7,8]. On the other hand, the Time-Frequency Analysis (TFA) methods [9,10], Wavelet Transform (WT) [11,12], and Hilbert–Huang Transform (HHT) [13,14] are successively applied to extract the transform domain characteristics from the received RF signals. However, these methods have little knowledge of impairments inside the individual emitter, and the performance can be easily affected by the wireless channels. Hence, there are many additional works in literature to model the characteristic of hardware imperfection of the internal component such as digital to analog converter (DAC) [15], modulator [16,17], and power amplifier (PA) [18,19,20,21], etc., and to extract the unique RF front-end feature for a particular emitter. This paper concentrates on the extraction of PA nonlinear features to identify communication emitters with multiple antennas under Multiple-Input Multiple-Output (MIMO) multipath channels.

It is well known that the MIMO transmission scheme can improve the spectral efficiency by introducing additional spatial diversity. In practice, transceivers with multiple transmit and receive antennas have shown their powerful merits and served as the major mechanism for current and future wireless communication systems. Meanwhile, due to the increasing number of antennas for MIMO emitters, there will be more diverse PA nonlinearities available at the RF front end for SEI. A lot of research efforts have been afforded to analyze the nonlinearity incurred by PAs with the Saleh model [22], polynomial model [23], and Volterra model [24], etc. in MIMO systems, whereas they are mainly devoted to implementing predistortion or PA linearization. However, to the authors’ best knowledge, there are few open results of SEI in MIMO communication systems. In [21], Li uses a modified artificial bee colony (ABC) algorithm to estimate the coefficients of the Hammerstein model, a simplified version of the Volterra model, in a MIMO system. However, the impact of wireless propagation channels is not considered, and the ABC algorithm also appears complicated to obtain the optimal solution in the context of SEI. Recently, in [25,26], an estimation of signal parameters via rotational invariance technique (ESPRIT)-based approach, which takes advantage of the multiple antennas at the receiver to separate the RFF from wireless channel, is proposed for RFF estimation in orthogonal frequency division multiplexing (OFDM) systems, whereas it is only suitable for a Single-Input Multiple-Output (SIMO) system rather than the MIMO one. In [19,20], a memoryless polynomial model is used to characterize the nonlinearity of PA, and a data-aided iterative algorithm is proposed to estimate nonlinear coefficients of the PA model for SEI from the observations in both MIMO and single-input single-output (SISO) scenarios.

Treating the fact that all PAs of a multiple-antenna emitter are independent from each other and following a memoryless polynomial model, in this paper, we propose two data-aided solutions that are different from the idea of [19,20] in the MIMO multipath scenario. Given received signals, we extend the method in [27] to the MIMO multipath scenario; a closed-form solution of the nonlinear coefficients is thus obtained by combining the linear least square (LLS) and singular value decomposition (SVD) methods. An alternative but more effective approach is also presented through solving a nonlinear least square (NLS) problem with independent variables consisting of both channel coefficients and nonlinear coefficients of the PA model. Furthermore, we explicitly provide deep discussion on the parameter estimate bias for the general case of unknown order of the PA model. In particular, it is proved in this paper that the rank-deficient property of both the NLS and LLS problems are in association with the amplitude level of the training sequence. The average misclassification rate based on the minimum error probability (MEP) criterion is theoretically derived, and we finally verify the proposed approaches via extensive numerical simulations.

The rest of the paper is organized as follows. In Section 2, we introduce the memoryless nonlinear model of PA in the MIMO multipath system. In Section 3, we present the linear and nonlinear frameworks for SEI, respectively. Then, the practical discussions for the proposed approaches and the misclassification rate are separately given in Section 4 and Section 5. In Section 6, numerical results are presented to demonstrate the effectiveness. Section 7 summarizes the paper.

## 2. Preliminaries and Problem Formulation

This paper mainly considers the scenario that *K* communication emitters equipped with multiple antennas are identified through a multiple-antenna receiver in the MIMO multipath environment. Since constant modulus modulation schemes such as phase-shift keying (PSK) may generally introduce less nonlinear distortion, we thus consider how to extract the underlying PA nonlinearity of each emitter from the received signal, with the help of a quadrature amplitude modulation (QAM) training sequence. In other words, PA nonlinearity is treated as a unique RFF of the emitter to fulfill the identification task, and we assume QAM is used by emitters when communicating with the receiver.

### 2.1. Memoryless Nonlinear PA Modeling

Generally, the memoryless polynomial model is simple in expression and can describe the intermodulation distortion of an RF PA well, such as PA9440 amplifier [28], in the narrowband communication system. More specifically, the polynomial coefficients are corresponding to the intermodulation coefficient such as third-order interception point ($IP_{3}$) and fifth-order interception point ($IP_{5}$). In this work, we choose the memoryless polynomial model to characterize the nonlinear behavior of all PA units in a multi-antenna emitter; then, the relationship between baseband equivalent input and output of the PA [20] at *j*th antenna branch can be written as

(1) $$x_{j}\left(n\right)=\sum_{p=1}^{(P+1)/2}\alpha_{2p-1,j}\cdot {\left(s_{j}\left(n\right)\right)}^{p}\cdot {\left(s_{j}^{*}\left(n\right)\right)}^{p-1},$$

where *P* denotes the max order of the PA model and can be configured as P=3,5,7,…. At the RF front-end of emitters, the frequency components that resulted from the even terms in the model can be removed by the bandpass filter, thus the even terms are ignored in Equation (1). $s_{j}\left(n\right)$ is the baseband equivalent input of the nonlinear system, denoting the *n*th QAM symbol transmitted over the *j*th antenna. The superscript “*” is the conjugate operator. $\alpha_{2p-1,j}$ denotes the normalized (2p−1)th order PA coefficient of the *j*th antenna and without loss of generality, we set $\alpha_{1,j}=1$ hereafter. $x_{j}\left(n\right)$ stands for the response of the nonlinear system.

In this paper, we suppose that each emitter is equipped with *J* antennas. Therefore, the normalized discrete-time baseband equivalent form of the nonlinear distortion model for the multi-antenna system can be expressed as

(2) $$\left[\begin{array}{c} x_{1}\left(n\right)\\ x_{2}\left(n\right)\\ \;\;\;\;\;\;\;\;\;\;\;\;\vdots \\ x_{J}\left(n\right) \end{array}\right]=\left[\begin{array}{c} {\bar{s}}_{1}\left(n\right)\\ {\bar{s}}_{2}\left(n\right)\\ \;\;\;\;\;\;\;\;\;\;\;\;\vdots \\ {\bar{s}}_{J}\left(n\right) \end{array}\right]+\sum_{p=2}^{(P+1)/2}\left[\begin{array}{cccc} \alpha_{2p-1,1} & 0 & \cdots & 0\\ 0 & \alpha_{2p-1,2} & \ldots & \vdots \\ \vdots & \vdots & \ddots & 0\\ 0 & \cdots & 0 & \alpha_{2p-1,J} \end{array}\right]\left[\begin{array}{c} {\left({\bar{s}}_{1}\left(n\right)\right)}^{p}\cdot {\left({\bar{s}}_{1}^{*}\left(n\right)\right)}^{p-1}\\ {\left({\bar{s}}_{2}\left(n\right)\right)}^{p}\cdot {\left({\bar{s}}_{2}^{*}\left(n\right)\right)}^{p-1}\\ \;\;\;\;\;\;\;\;\;\;\;\;\;\;\;\;\;\;\;\;\;\;\;\;\;\;\;\;\;\;\;\;\;\;\;\;\;\;\;\;\;\;\;\;\;\vdots \\ {\left({\bar{s}}_{J}\left(n\right)\right)}^{p}\cdot {\left({\bar{s}}_{J}^{*}\left(n\right)\right)}^{p-1} \end{array}\right],$$

where ${\bar{s}}_{j}\left(n\right)$ is the normalized version of $s_{j}\left(n\right)$.

### 2.2. MIMO Multipath Channel

The propagation channel considered in this paper is a linear discrete MIMO system with *J* transmit antennas and *R* receive antennas. Given the length of the training sequence *N*, the signal received at the antenna *r* can be represented by

(3) $$y_{r}=h_{r1}⊗x_{1}+h_{r2}⊗x_{2}+\cdots +h_{rJ}⊗x_{J}+v_{r},$$

where ⊗ denotes the convolution operation. $x_{j}$ is the nonlinear distortion signal transmitted over the *j*th antenna, $h_{rj}$ represents the channel impulse response between the *j*th transmit antenna to the *r*th receive antenna and remains time-invariant during the data receiving process, and $v_{r}$ is the zero-mean additive white Gaussian noise received at the antenna *r*. Thus, we can unfold Equation (3) naturally in the form of

(4) $$y_{r}=X^{\left(1\right)}h_{r1}+X^{\left(2\right)}h_{r2}+\ldots +X^{\left(J\right)}h_{rJ}+v_{r},$$

where the order of the channel is *L*, $X^{\left(j\right)}\in C^{(N+L-1)\times L}$ is a Toeplitz matrix populated by $x_{j}$ in Equation (3). Note that the nonlinear coefficients are cross-coupled with channel coefficients in Equation (4); therefore, the major target arising in this paper is to get the accurate estimations of the separate nonlinear coefficient $\left\{\alpha_{2p-1,j}\right\}$ via the received signal $\left\{y_{r}\right\}$ with the aid of training sequences in MIMO multipath scenarios, before we can identify a specific emitter.

## 3. The Proposed Estimation Approaches

As mentioned above, it has been reported in [19,20] that the nonlinear coefficients of PAs can be estimated with two stages. The first stage establishes the initial estimation of channel coefficients and nonlinear coefficients through some well-designed training sequences sorted by amplitude. Then, an iterative method is applied at the second stage to eliminate the estimate bias. In [27], a PA parameter estimator combined the best linear unbiased estimation (BLUE) and singular value decomposition (SVD) is proposed for the SISO system. Since the method in [27] can get a closed-form solution of the PA nonlinear coefficients and has no constraint on the ordering of the amplitude of training symbols, which is more practical compared to the one in [19], we extend it to the MIMO multi-path scenarios and mark it as linear method in MIMO (LMM). Furthermore, we propose an alternative method to extract PA parameters in a nonlinear least square (NLS) manner. It should be noted that both LMM and NLS approaches can be degraded into SISO systems.

### 3.1. The LMM Approach

Note that, in [27], if the product terms of the channel coefficients and the nonlinear coefficients are substituted by some new integrated variables, we can obtain a set of linear equations with regard to the new variables, thus the product terms can be readily solved by LLS. Then, the only thing left to us is to extract $\alpha_{2p-1,j}$ from the solution.

According to Equation (4), we first vectorize signals received by all *R* antennas into vector $y_{vec}$, i.e., $y_{vec}={\left[y_{1}^{T},y_{2}^{T},\ldots ,y_{R}^{T}\right]}^{T}\in C^{R(N+L-1)}$, where the superscript “T” is the transpose operator. In addition, the signal at the receiver side now is

(5) $$y_{vec}=D_{s}h_{\alpha }+v_{vec},$$

where $v_{vec}$ is the corresponding reshaped noise vector. $h_{\alpha }\in C^{RJL(P+1)/2}$ is the integrated vector composed of all independent variables and can be represented as:

(6) $$h_{\alpha }={\left[{u_{1,0}}^{T},\ldots ,{u_{1,L-1}}^{T}\left|\ldots \left|{u_{R,0}}^{T},\ldots ,{u_{R,L-1}}^{T}\right.\right.\right]}^{T},$$

with

(7) $$u_{r,l_{h}}=W_{r,l_{h}}\alpha_{vec},$$

(8) $$\alpha_{j}={\left[1,\alpha_{3,j},\alpha_{5,j},\ldots ,\alpha_{P,j}\right]}^{T},$$

(9) $$\alpha_{vec}={\left[{\alpha_{1}}^{T},{\alpha_{2}}^{T},\ldots ,{\alpha_{J}}^{T}\right]}^{T},$$

(10) $$W_{r,l_{h}}=blkdiag\left(h_{r1}\left(l_{h}\right)I_{\alpha },h_{r2}\left(l_{h}\right)I_{\alpha },\ldots ,h_{rJ}\left(l_{h}\right)I_{\alpha }\right),$$

in which $l_{h}=0,\ldots ,L-1$, $I_{\alpha }\in C^{(P+1)/2\times (P+1)/2}$ is a unit matrix and blkdiag(·) is the block diagonalization function. In addition, $D_{s}$ is defined by

(11) $$D_{s}=blkdiag\left(\underset{R}{\underset{︸}{d_{s},d_{s},\ldots ,d_{s}}}\right),$$

with $d_{s}\in C^{(N+L-1)\times JL(P+1)/2}$ being constructed by the known training sequence. To elaborate the process, we provide a numerical example in Appendix A.

Consequently, the least square (LS) estimation of $h_{\alpha }$ in Equation (5) is

(12) $${\hat{h}}_{\alpha }=D_{s}^{†}y_{vec},$$

where the superscript “†” denotes the pseudo-inverse operation. It is worth noting that, compared to the BLUE method in [27], the least square solution of Equation (12) has no requirement on the estimation of noise power. Obviously, the condition of a unique solution to Equation (12) is natural that $D_{s}$ is full column rank and N≥JL(P+1)/2−L+1 is satisfied.

Afterwards, we define

(13) $$Q=\left[{\hat{u}}_{1,0},\ldots ,{\hat{u}}_{1,L-1}\left|\ldots \left|{\hat{u}}_{R,0},\ldots ,{\hat{u}}_{R,L-1}\right.\right.\right]$$

and further perform SVD on the matrix $Q\in C^{J(P+1)/2\times RL}$ to get a closed-form estimation of the PA parameters in the MIMO system. Since the normalized first-order nonlinear coefficients are assumed equal to 1, the execution steps of PA nonlinear coefficients estimator can be summarized as:

(1) Reshape the observations according to Equation (5), and estimate $h_{\alpha }$ according to Equation (12).
(2) Reshape the ${\hat{h}}_{\alpha }$ into the matrix Q, then perform SVD on $Q_{j}=U_{j}\sum_{j}V_{j}^{H}$, where $Q_{j}$ is a submatrix consisting of the $\left((j-1)(P+1)/2+1\right)$th to $\left(j(P+1)/2\right)$th rows of the matrix Q.
(3) Estimate the nonlinear coefficients of *j*th transmitting antenna as follows:
  (14) $${\hat{\alpha }}_{j}=\frac{1}{U_{j}^{\left(1,1\right)}}U_{j}^{\left(:,1\right)},$$
  where $U_{j}^{\left(:,1\right)}$ and $U_{j}^{\left(1,1\right)}$ are the first column and first element of $U_{j}$, respectively.

### 3.2. The NLS Approach

Bearing in mind the received signal in Equation (4) for MIMO multipath transmission system, we can now further expand the expression with a series of nonlinear equations due to the existence of product term of the nonlinear coefficient $\alpha_{2p-1,j}$ and the channel coefficient $h_{rj}\left(l_{h}\right)$. As a consequence, the problem of nonlinear coefficients estimation can thus be alternatively transformed into a NLS optimization one when introducing a training sequence with length *N*.

In order to get a more robust solution, we choose to solve a constrained NLS optimization problem with $q=RJL+J\left(P-1\right)/2$ independent variables, and the cost function can be given by:

(15) $$\underset{z}{min}G_{\gamma }\left(z\right)=\underset{z}{min}\frac{1}{2}{∥g(z)∥}_{2}^{2}+\frac{1}{2}{∥\gamma z∥}_{2}^{2},$$

where ${∥\cdot ∥}_{2}$ denotes 2-norm. $z\in C^{q}$ is the vector consisting of the independent variables and can be described as $z={\left[z_{h}^{T},z_{\alpha }^{T}\right]}^{T}$, in which $z_{\alpha }$ and $z_{h}$ are respectively as

(16) $$z_{\alpha }={\left[\alpha_{3,1},\ldots ,\alpha_{P,1},\ldots ,\alpha_{3,J},\ldots ,\alpha_{P,J}\right]}^{T}$$

(17) $$z_{h}={\left[{H_{1}}^{T},{H_{2}}^{T},\ldots ,{H_{R}}^{T}\right]}^{T},$$

with

(18) $$H_{r}={\left[h_{r1}\left(0\right),\ldots ,h_{rJ}\left(0\right),\ldots ,h_{r1}\left(L-1\right),\ldots ,h_{rJ}\left(L-1\right)\right]}^{T}.$$

γz22γz2222 is the regularization. $g\left(z\right)\in C^{R(N+L-1)}$ denotes the residual function: $C^{q}\to C^{R(N+L-1)}$ with R(N+L−1)≥q, that is,

(19) $$g\left(z\right)={\left[\Delta {y_{1}}^{T},\Delta {y_{2}}^{T},\ldots ,\Delta {y_{R}}^{T}\right]}^{T},$$

with

(20) $$\Delta y_{r}=y_{r}-\left(X^{\left(1\right)}h_{r1}+X^{\left(2\right)}h_{r2}+\ldots +X^{\left(J\right)}h_{rJ}\right).$$

According to [29], the regularized Gauss–Newton iterative method is introduced here to figure out z, i.e.,

(21) $$z_{i+1}=z_{i}+\Delta z_{i}$$

with

(22) $$\Delta z_{i}=-{\left({\left(J(z_{i})\right)}^{H}J\left(z_{i}\right)+\gamma^{2}I_{q}\right)}^{-1}{\left[\begin{array}{c} J(z_{i})\\ \gamma I_{q} \end{array}\right]}^{H}\left[\begin{array}{c} g(z_{i})\\ \gamma z_{i} \end{array}\right].$$

The superscript “*H*” denotes conjugate transposition, and $I_{q}$ is a q×q unit matrix. J(z) is the Jacobian matrix of g(z), that is, the first-order derivative of g(z) with respect to z.

In general, given the Jacobian matrix J(z) with full column rank, one can apply the regularized Gauss–Newton method in Equation (21) to optimize problem Equation (15) and can eventually obtain the optimal solution $z_{opt}$. In addition, the appropriate regularization factor γ can improve the condition number of the inverse matrix in Equation (22), which guarantees the robustness of the NLS optimization.

## 4. Practical Discussions on the Proposed Approaches

As mentioned in Section 3, we can get a closed-form solution for the problem of nonlinear coefficient estimation through the LMM approach. Alternatively, an iterative NLS approach can also be applicable to extract PA parameters from observations. However, in practice, there are two main factors that affect the accuracy of our proposed algorithms. The first factor is the case where the order of PA model is mismatched between the transmitting and receiving ends. The second one is the case where the matrix $D_{s}$ and the Jacobian matrix J(z) are rank-deficient. In the next Section 4.1 and Section 4.2, we first theoretically analyze the impact of the mismatched model. After that, we present the rank-deficient condition of the $D_{s}$ and J(z) matrix in Section 4.3.

### 4.1. Overdetermined Order of the PA Model

In this subsection, we consider the impact of the overdetermined order of the PA model on the estimation accuracy of the nonlinear coefficients. Assume that all the PAs of an emitter actually have the same model order as *P*. If we obtain an overdetermined order of the PA model beforehand, e.g., $P_{0}$ with $\left(P_{0}>P\right)$, the expected observation of $y_{vec}=D_{s}^{\left(1,\ldots ,P\right)}h_{\alpha }^{\left(1,\ldots ,P\right)}+v_{vec}$ may be formulated as $y_{vec}=D_{s}^{\left(1,\ldots ,P_{0}\right)}h_{\alpha }^{\left(1,\ldots ,P_{0}\right)}+v_{vec}$ instead. Since $D_{s}^{\left(1,\ldots ,P\right)}h_{\alpha }^{\left(1,\ldots ,P\right)}$ equals $\left(D_{s}^{\left(1,\ldots ,P_{0}\right)}h_{\alpha }^{\left(1,\ldots ,P_{0}\right)}-D_{s}^{\left(P+2,\ldots ,P_{0}\right)}h_{\alpha }^{\left(P+2,\ldots ,P_{0}\right)}\right)$, the estimation of $h_{\alpha }$ can thus be expressed as follows:

(23) $$\begin{array}{cc} {\hat{h}}_{\alpha } & ={\left(D_{s}^{\left(1,\ldots ,P_{0}\right)}\right)}^{†}y_{vec}\\ & ={\left(D_{s}^{\left(1,\ldots ,P_{0}\right)}\right)}^{†}\left(D_{s}^{\left(1,\ldots ,P\right)}h_{\alpha }^{\left(1,\ldots ,P\right)}+v_{vec}\right)\\ & =h_{\alpha }^{\left(1,\ldots ,P_{0}\right)}-{\left(D_{s}^{\left(1,\ldots ,P_{0}\right)}\right)}^{†}\left(D_{s}^{\left(P+2,\ldots ,P_{0}\right)}h_{\alpha }^{\left(P+2,\ldots ,P_{0}\right)}\right)+{\left(D_{s}^{\left(1,\ldots ,P_{0}\right)}\right)}^{†}v_{vec}, \end{array}$$

where $D_{s}^{\left(a,\ldots ,b\right)}$ denotes a matrix $D_{s}$ that is populated by the *a*th to the *b*th order versions of training sequence, and $h_{\alpha }^{\left(a,\ldots ,b\right)}$ denotes a vector $h_{\alpha }$ that is populated by the corresponding *a*th to the *b*th order nonlinear coefficients. Some numerical examples for the construction process are also provided in Appendix A.

Note that the (P+2)th to the $P_{0}$th order the nonlinear coefficients and $h_{\alpha }^{\left(P+2,\ldots ,P_{0}\right)}$ should be all zeros in this case, then, we have ${\hat{h}}_{\alpha }=h_{\alpha }^{\left(1,\ldots ,P_{0}\right)}+{\left(D_{s}^{\left(1,\ldots ,P_{0}\right)}\right)}^{†}v_{vec}$, which is equivalent to the unbiased estimation of the $h_{\alpha }^{\left(1,\ldots ,P\right)}$, and the additive white noise has no effect on the unbiasedness of the estimation results. Therefore, we can conclude that, if the order of the PA model is overdetermined, the estimation of the nonlinear coefficients obtained by the LMM approach is still unbiased.

### 4.2. Underdetermined Order of the PA Model

In the sequel, we attempt to analyze the case that the order of the PA model is underdetermined, i.e., $P_{0}$ is lower than the actual *P*. It is easy to understand that $D_{s}^{\left(1,\ldots ,P\right)}h_{\alpha }^{\left(1,\ldots ,P\right)}$ now equals $\left(D_{s}^{\left(1,\ldots ,P_{0}\right)}h_{\alpha }^{\left(1,\ldots ,P_{0}\right)}+D_{s}^{\left(P_{0}+2,\ldots ,P\right)}h_{\alpha }^{\left(P_{0}+2,\ldots ,P\right)}\right)$, so that the estimation of $h_{\alpha }$ can be written as:

(24) $$\begin{array}{c} {\hat{h}}_{\alpha }=h_{\alpha }^{\left(1,\ldots ,P_{0}\right)}+{\left(D_{s}^{\left(1,\ldots ,P_{0}\right)}\right)}^{†}\left(D_{s}^{\left(P_{0}+2,\ldots ,P\right)}h_{\alpha }^{\left(P_{0}+2,\ldots ,P\right)}\right)+{\left(D_{s}^{\left(1,\ldots ,P_{0}\right)}\right)}^{†}v_{vec}. \end{array}$$

Attention must be paid to non-zero $h_{\alpha }^{\left(P_{0}+2,\ldots ,P\right)}$ in Equation (24) due to the non-zero nonlinear PA coefficients through the $\left(P_{0}+2\right)$th to the *P*th order. The resulting ${\hat{h}}_{\alpha }$ is a biased estimation w.r.t. $h_{\alpha }^{\left(1,\ldots ,P\right)}$ and the estimate bias equals ${\left(D_{s}^{\left(1,\ldots ,P_{0}\right)}\right)}^{†}\left(D_{s}^{\left(P_{0}+2,\ldots ,P\right)}h_{\alpha }^{\left(P_{0}+2,\ldots ,P\right)}\right)$.

Furthermore, if we define a guess as:

**Guess** **1.** *If the training squences are discrete amplitude communication symbols such as PSK and QAM, then*

(25) $${\left(D_{s}^{\left(1,\ldots ,P_{0}\right)}\right)}^{†}D_{s}^{\left(P_{0}+2,\ldots ,P\right)}=blkdiag\left(\underset{RJL}{\underset{︸}{c,c,\ldots ,c}}\right),$$

*where*$c\in C^{m\times n}$*is a constant matrix with*$m=\frac{P_{0}+1}{2}$*and*$n=\frac{P-P_{0}}{2}$*.*

Then, the estimate bias of the nonlinear PA coefficients can be given as the following Proposition 1.

**Proposition** **1.** *If Guess 1 holds true, the estimate bias of the nonlinear PA coefficients obtained by SVD according to Equation (14) is shown as*

(26a)Δα3,j=(c21αP0+2,j+…+c2nαP,j)−(c11αP0+2,j+…+c1nαP,j)α3,j1+(c11αP0+2,j+…+c1nαP,j)(26b)⋮(26c)ΔαP0,j=(cm1αP0+2,j+…+cmnαP,j)−(cm1αP0+2,j+…+cmnαP,j)αP0,j1+(c11αP0+2,j+…+c1nαP,j).

**Proof** **of** **Proposition** **1.** See Appendix B. □

Therefore, we can conclude that, when the order of the PA model is underdetermined, the bias term of each nonlinear coefficient is related to training sequence and the higher-order nonlinear coefficients. In addition, the high-order nonlinear coefficients (i.e., $\alpha_{P_{0}+2,j}$ to $\alpha_{P,j}$) are still unknown.

### 4.3. Rank Deficiency Condition of the Proposed Approaches

As mentioned earlier, it is impossible to determine a unique solution for the LLS problem in Equation (12) when the matrix $D_{s}$ is rank-deficient. In addition, for the NLS problem in Equation (15), it is also hard for the regularized Gauss–Newton method in Equation (21) to find the global optimal solution when the Jacobian matrix J(z) is rank-deficient at every z. However, since QAM is employed by all emitters, the training sequence has only a limited level of amplitude. We find that the rank attributes of $D_{s}$ and J(z) are both associated with the maximum order of the PA model. The specific relationship can be revealed by Proposition 2 as follows.

**Proposition** **2.** Given QAM symbols with M modulus values as the training sequence and the maximum P order of the PA model, the matrix $D_{s}$ is rank-deficient and the Jacobian matrix J(z) is also rank-deficient at every z, if M<(P+1)/2.

**Proof** **of** **Proposition 2.** See Appendix C. □

According to the Proposition 2, the $D_{s}$ and J(z) are full rank as long as the amplitude type of signal is sufficient. Therefore, our proposed approach can be applied in some single-carrier communication systems with higher order QAM modulation such as 16-QAM, 64-QAM and 256-QAM, which have 3, 9, and 32 different modulus values, respectively. Actually, it appears that our proposed approaches are readily suitable for other popular wireless communication systems, such as MIMO-OFDM systems, where there will be no rank-deficient problem of $D_{s}$ and J(z) in nature since the amplitude of the transmitted signal is generally continuous. Moreover, it is not necessary to estimate the channel order in a MIMO-OFDM system due to its ability to resist multipath effects, which will make the proposed approach more practical.

## 5. Error Rate Analysis For Classification

In this paper, we apply a minimum error probability (MEP) criterion [30] based on Bayesian theory to classify different emitters, and the RFF feature of each emitter is composed of the estimated nonlinear coefficients and can be expressed as follows:

(27) $$a={\left[{a_{3}}^{T},{a_{5}}^{T},\ldots ,{a_{P}}^{T}\right]}^{T},$$

with

(28) $$a_{p}={\left[Re\left({\hat{\alpha }}_{p,1}\right),\ldots ,Re\left({\hat{\alpha }}_{p,J}\right),Im\left({\hat{\alpha }}_{p,1}\right),\ldots ,Im\left({\hat{\alpha }}_{p,J}\right)\right]}^{T},$$

where p=3,5,…,P.

Here, we take the case where there are two emitters as an example to give the derivation of the decision criteria, and the binary hypothesis test model can be considered as

(29a)C1:a=m1+vα,(29b)C2:a=m2+vα,

where $C_{i}$ denotes the category *i*, i=1,2; $v_{\alpha }$ is composed of the residual additive Gaussian noise in the estimations of the nonlinear coefficients and we assume that each element of $v_{\alpha }$ obeys a Gaussian distribution with a zero mean and a variance of $\delta^{2}$; $m_{1}\in C^{J(P-1)}$ and $m_{2}\in C^{J(P-1)}$ are respectively the mean vectors of the estimated feature vector a for the two emitters, which can be obtained from the samples collected offline. Thus, the decision rule can be derived based on MEP criterion as follows:

(30a)(m2−m1)Ta>m2Tm2−m1Tm12,a∈C2,(30b)(m2−m1)Ta<m2Tm2−m1Tm12,a∈C1,

where the test statistic is $a_{ts}={\left(m_{2}-m_{1}\right)}^{T}a$ and the decision threshold is thr=(m2Tm2−m1Tm1)(m2Tm2−m1Tm1)22.

For simplicity, if we assume that the variables in a are independent of each other, then the test statistic $a_{ts}∼N\left(m_{ts},\delta_{ts}^{2}\right)$, for the *i*-th emitter, the mean is as $m_{ts}^{\left(i\right)}={\left(m_{2}-m_{1}\right)}^{T}m_{i},i=1,2$, and the variance is as $\delta_{ts}^{2}=\sum_{k=1}^{J(P-1)}\delta^{2}\beta_{k}^{2}$ with $\beta_{k}$ being the *k*-th element of $\left(m_{2}-m_{1}\right)$. As a result, with the assumption of equally probable hypotheses, the average misclassification rate based on MEP criterion can be derived as

(31) $$\begin{array}{cc} {\bar{P}}_{e} & =\frac{1}{2}\left[P_{r}\left(\left.a_{ts}>thr\right|C_{1}\right)+P_{r}\left(\left.a_{ts}<thr\right|C_{2}\right)\right]\\ & =\Phi \left(-\frac{{\left(m_{2}-m_{1}\right)}^{T}\left(m_{2}-m_{1}\right)}{2\delta_{ts}}\right)\\ & =\Phi \left(-\frac{\sqrt{{\left(m_{2}-m_{1}\right)}^{T}\left(m_{2}-m_{1}\right)}}{2\delta }\right), \end{array}$$

where $\Phi \left(x\right)=\int_{-\infty }^{x}\frac{1}{\sqrt{2\pi }}e^{-x^{2}/2}dx$ and Φ(−x)=1−Φ(x). When *x* increases, Φ(−x) decreases.

As for the case where the estimations of the nonlinear coefficients are unbiased, the mean values of the RFF feature for the two emitters are readily as

(32) $$m_{i}={\left[{{m_{i}}^{\left(3\right)}}^{T},{{m_{i}}^{\left(5\right)}}^{T},\ldots ,{{m_{i}}^{\left(P\right)}}^{T}\right]}^{T}$$

with

(33) $${m_{i}}^{\left(p\right)}={\left[Re\left(\alpha_{p,1}^{\left(i\right)}\right),\ldots ,Re\left(\alpha_{p,J}^{\left(i\right)}\right),Im\left(\alpha_{p,1}^{\left(i\right)}\right),\ldots ,Im\left(\alpha_{p,J}^{\left(i\right)}\right)\right]}^{T},$$

where $\alpha_{p,j}^{\left(i\right)}$ is the nonlinear coefficient of the *i*-th emitter. However, for the case of biased estimations, ${\tilde{m}}_{i}=m_{i}+\Delta m_{i}$, where

(34) $$\begin{array}{c} \Delta m_{i}={\left[{\Delta {m_{i}}^{\left(3\right)}}^{T},{\Delta {m_{i}}^{\left(5\right)}}^{T},\ldots ,{\Delta {m_{i}}^{\left(P\right)}}^{T}\right]}^{T} \end{array}$$

with

(34) $$\begin{array}{c} \Delta {m_{i}}^{\left(p\right)}={\left[Re\left(\left[\Delta \alpha_{p,1}^{\left(i\right)},\ldots ,\Delta \alpha_{p,J}^{\left(i\right)}\right]\right),Im\left(\left[\Delta \alpha_{p,1}^{\left(i\right)},\ldots ,\Delta \alpha_{p,J}^{\left(i\right)}\right]\right)\right]}^{T} \end{array}$$

being the mean of the biases with i=1,2 in the estimation of RF fingerprint features. Therefore, according to Equation (31), the average misclassification rate for this case can be obtained by:

(36) $$\begin{array}{cc} {\bar{P}}_{e} & =\Phi \left(-\frac{{\left({\tilde{m}}_{2}-{\tilde{m}}_{1}\right)}^{T}\left({\tilde{m}}_{2}-{\tilde{m}}_{1}\right)}{2\sqrt{\sum_{k=1}^{J(P-1)}\delta^{2}{\tilde{\beta }}_{k}^{2}}}\right)\\ & =\Phi \left(-\frac{\sqrt{\theta_{1}+\theta_{2}+\theta_{3}}}{2\delta }\right), \end{array}$$

where $\Delta \beta_{k}$ is the *k*-th element of $\left(\Delta m_{2}-\Delta m_{1}\right)$ and

{(37a)θ1=(m2−m1)T(m2−m1)(37b)θ2=2(m2−m1)T(Δm2−Δm1)(37c)θ3=(Δm2−Δm1)T(Δm2−Δm1).

Therefore, we can conclude as follows:

(1) The ${\bar{P}}_{e}$ decreases as the variance of the additive noise decrease.
(2) The ${\bar{P}}_{e}$ decreases as the difference between the mean values of the RF fingerprint feature for the two emitters increases, which indicates that the PA parameters of each emitter should be designed to be as different as possible in a bid to achieve better performance.
(3) According to Equation (31), as for a fixed *P*, more nonlinear coefficients are used as features will make the $\sqrt{{\left(m_{2}-m_{1}\right)}^{T}\left(m_{2}-m_{1}\right)}$ larger, which obviously leads to better classification performance.
(4) According to Equation (36), when there are biases in the estimations, the additional terms $\theta_{2}$ and $\theta_{3}$ may cause ${\bar{P}}_{e}$ to decrease compared to the case where there is no bias.

More generally, as for K (K>2) emitters, with the assumption of equally probable hypotheses, the average misclassification rate based on MEP criterion can be represented as

(38) $${\bar{P}}_{e}=1-{\bar{P}}_{c}=1-\frac{1}{K}\left[P_{r}\left(D_{1}\left|C_{1}\right.\right)+P_{r}\left(D_{2}\left|C_{2}\right.\right)+\cdots +P_{r}\left(D_{K}\left|C_{K}\right.\right)\right],$$

where ${\bar{P}}_{c}$ is the average correct classification rate; $D_{k}\left(k=1,2,\ldots ,K\right)$ indicates the discriminant domain of the kth class and it shows as Equation (39)

(39) mk−m1Ta>mkTmk−m1Tm1mkTmk−m1Tm122⋮mk−mk−1Ta>mkTmk−mk−1Tmk−1mkTmk−mk−1Tmk−122mk−mk+1Ta>mkTmk−mk+1Tmk+1mkTmk−mk+1Tmk+122⋮mk−mKTa>mkTmk−mKTmKmkTmk−mKTmK22.

Therefore, the $P_{r}\left(D_{k}\left|C_{k}\right.\right)$ can be obtained by solving the multiple integrals in Equation (40)

(40) $$\begin{array}{c} P_{r}\left(D_{k}\left|C_{k}\right.\right)\\ =\int_{\frac{m_{k}^{T}m_{k}-m_{K}^{T}m_{K}}{2}}^{+\infty }\cdots \int_{\frac{m_{k}^{T}m_{k}-m_{1}^{T}m_{1}}{2}}^{+\infty }f\left(w_{1}\right)\ldots f\left(w_{k-1}\right)f\left(w_{k+1}\right)\ldots f\left(w_{K}\right)d_{w_{1}}\ldots d_{w_{k-1}}d_{w_{k+1}}\ldots d_{w_{K}}, \end{array}$$

where $f\left(w_{i}\right)=\frac{1}{\sqrt{2\pi }\delta_{w_{i}}}\exp\left(-\frac{{\left(w_{i}-{\left(m_{k}-m_{i}\right)}^{T}m_{k}\right)}^{2}}{2\delta_{w_{i}}^{2}}\right)$ with $w_{i}={\left(m_{k}-m_{i}\right)}^{T}a$ (i=1,2,…,K and i≠k), and $\delta_{w_{i}}$ is a standard deviation similar to the $\delta_{ts}$ in Equation (31).

## 6. Numerical Results

### 6.1. Simulation Setting

In the following simulations, if not specified, we use a 2×2 MIMO channel with Rayleigh multipath fading model, and the number of paths *L* is set to 2. The training sequences are QAM symbols with N=35 for proper complexity and performance trade-off. The regularization factor γ is set to 0.05 for the trade-off between the variance and bias in the estimation of NLS approach. The complex nonlinear coefficients lead to AM–AM and AM–PM distortion, where the AM–AM distortion is mainly caused by the $IP_{3}$ and $IP_{5}$, and higher order intermodulation distortion is usually ignored [28,31]. Generally, the actual communication system has specific requirements for the out-of-band spectral emission level of RF signals. Therefore, for the rationality of PA parameters in reality, we select the PA parameters that AM–AM characteristic obeys the method in [28,31] and the AM–PM is obtained by slightly adjusting the phase of the parameter in [20]. The nonlinear PA coefficients are displayed in Table 1, and it has been verified that the out-of-band emission level of the amplified signals is about 40 dBr, which meets the requirement of general agreement.

In all experiments, we use the Normalized Mean Squared Error (NMSE) to evaluate the estimation accuracy of the nonlinear coefficients, i.e.,

(41) $$err_{\alpha }=10log10\left(E\left\{\frac{{∥\;\alpha_{est}-\alpha_{true}∥}_{2}^{2}}{{∥\;\alpha_{true}∥}_{2}^{2}}\right\}\right),$$

where $\alpha_{true}={\left[\alpha_{3,1},\alpha_{3,2},\alpha_{5,1},\alpha_{5,2},\ldots ,\alpha_{P,1},\alpha_{P,2}\right]}^{T}$, $\alpha_{est}$ is the estimation of the $\alpha_{true}$, and E{·} denotes the Mathematical Expectation.

### 6.2. Simulation Results

#### 6.2.1. The Impact of SNR

**Figure 1:**
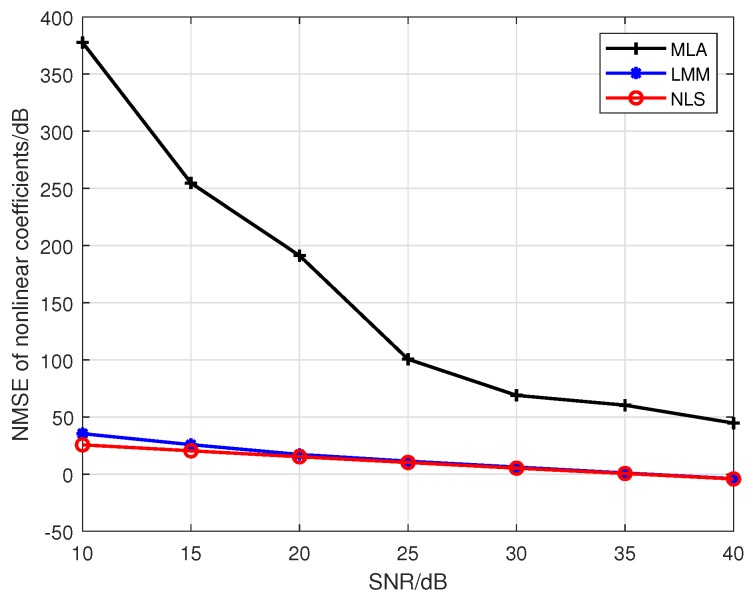
The comparison of the estimation accuracy among the MLA, LMM, and NLS.

**Figure 2:**
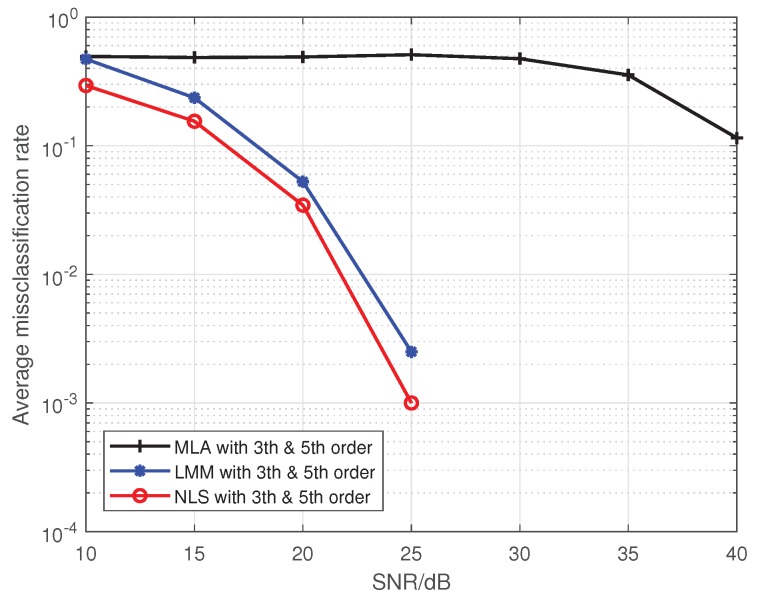
The comparison of the average misclassification rate among the MLA, LMM, and NLS.

**Figure 3:**
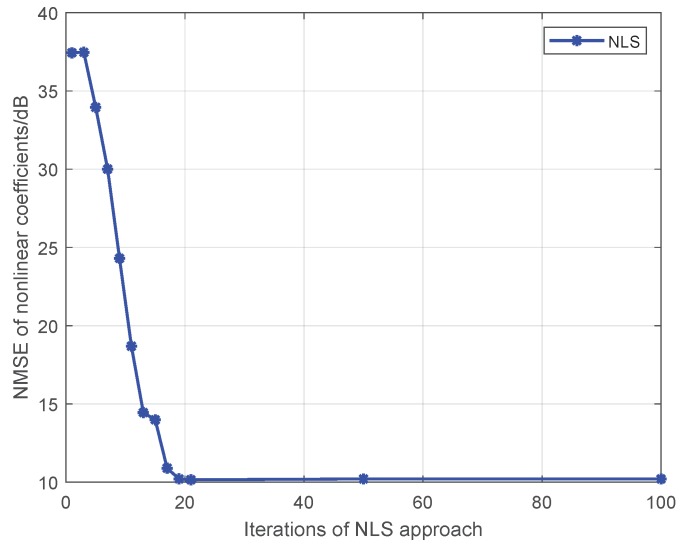
The impact of iterations on the estimation accuracy for the NLS approach.

**Figure 4:**
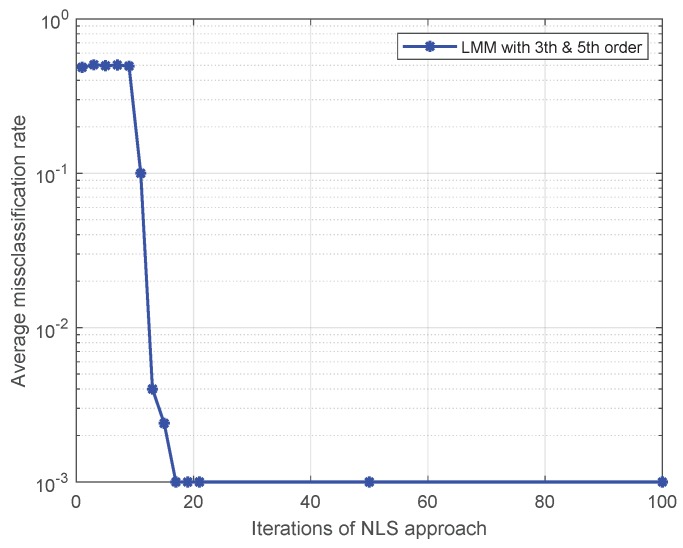
The impact of iterations on the average misclassification rate for the NLS approach.

Note that Liu’s algorithm in [19] assumes all PAs in one emitter are the same. To facilitate the comparison of the proposed approaches with Liu’s algorithm, we extend Liu’s algorithm to be suitable for our MIMO scenario. For brevity, we use the name Modified Liu Algorithm (MLA) to represent the modified version in the following. Here, we set *P* to 5, and the training sequence is 16-QAM symbols. At first, we give comparisons among the MLA, LMM, and NLS in Figure 1 and Figure 2, where the third-order and fifth-order coefficients are combined as a classification feature, from which one can see that the performance of the LMM and NLS are apparently better than that of MLA, especially at low signal to noise ratio (SNR) regime. One possible reason lies in that when *P* equals 5 and the training sequence is composed of 16-QAM symbols, the $D_{s}$ and J(z) are both full column rank, whereas the matrix populated by the training sequence for the initial estimation of MLA method is rank-deficient regardless of how long the training sequence is. In addition, we notice that the NLS approach also performs better than the LMM one, which is because the introduction of regularization during the process of optimization. Furthermore, in order to explore the impact of iterations on performance of NLS approach, when SNR is 25 dB, we give some results in Figure 3 and Figure 4 to demonstrate the convergence speed of NLS approach, where 19 iterations are needed to obtain the optimal solution.

**Figure 5:**
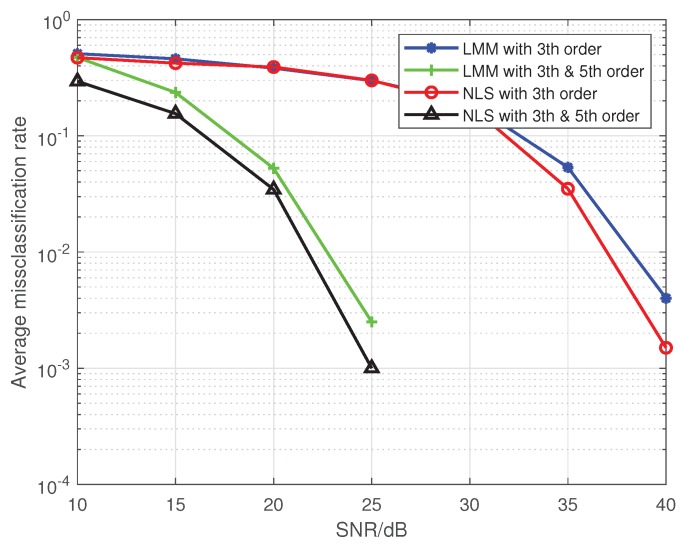
The impact of SNR on the average misclassification rate for two emitters.

In Figure 5, we fit a PA with P=5, when third-order and fifth-order coefficients are combined as a classification feature, the classification performance is better than when only the third-order coefficient is used. This confirms the analysis that, for a fixed *P*, features with more nonlinear coefficients can effectively improve classification performance in Section 5. In the next simulations, if not specified, we use the estimated third-order and fifth-order coefficients together as classification features.

**Figure 6:**
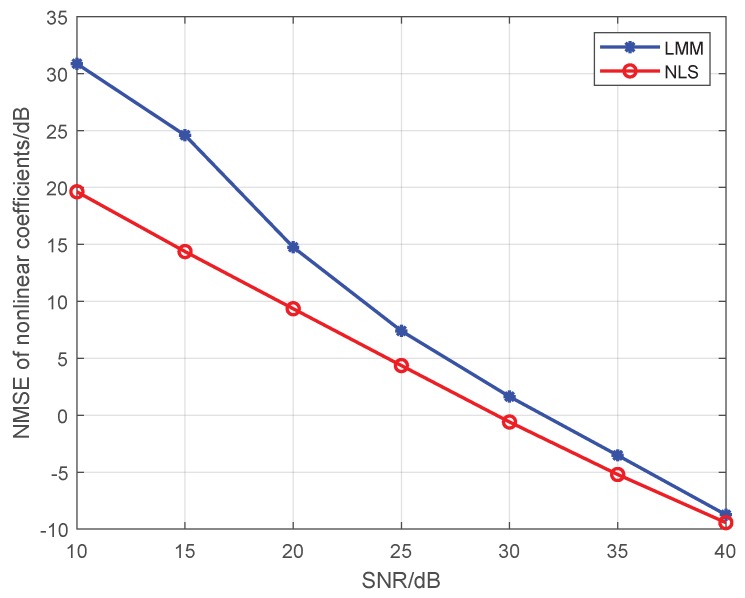
The impact of SNR on the estimation accuracy under a 4×4 MIMO scenario.

**Figure 7:**
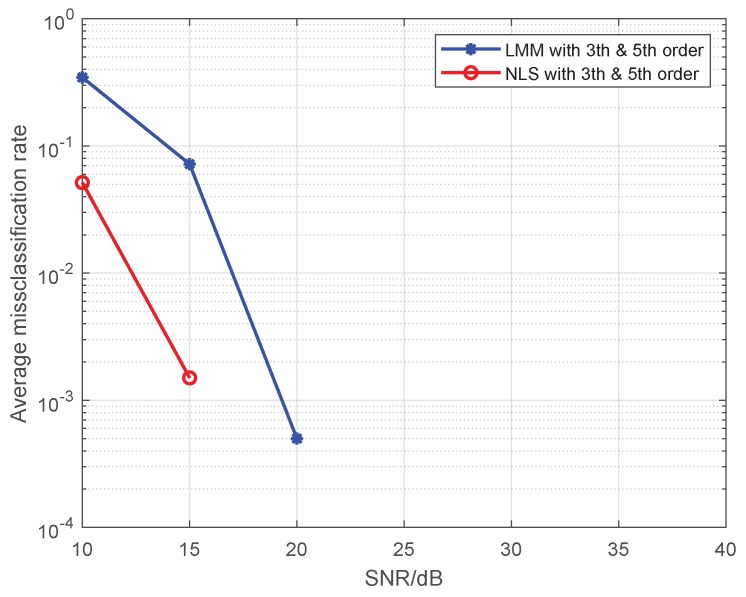
The impact of SNR on the average misclassification rate under a 4×4 MIMO scenario.

Moreover, in Figure 6 and Figure 7, we present results of the proposed approaches under a 4×4 MIMO scenario; as you can see, more RF channels bring more nonlinear coefficients, which is beneficial to improve classification performance. Here, for convenience, we take the nonlinear coefficients of Emitter2 and Emitter4 in Table 1 as those of the first 4-antenna emitter, and take the nonlinear coefficients of Emitter3 and Emitter6 in Table 1 as those of the second 4-antenna emitter.

#### 6.2.2. Identification for Multiple Emitters

**Figure 8:**
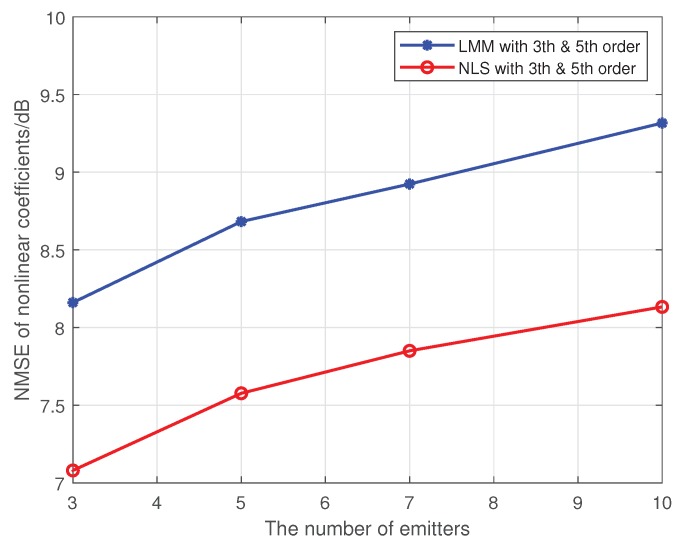
The impact of the number of emitters on the estimation accuracy for multiple transmitters.

**Figure 9:**
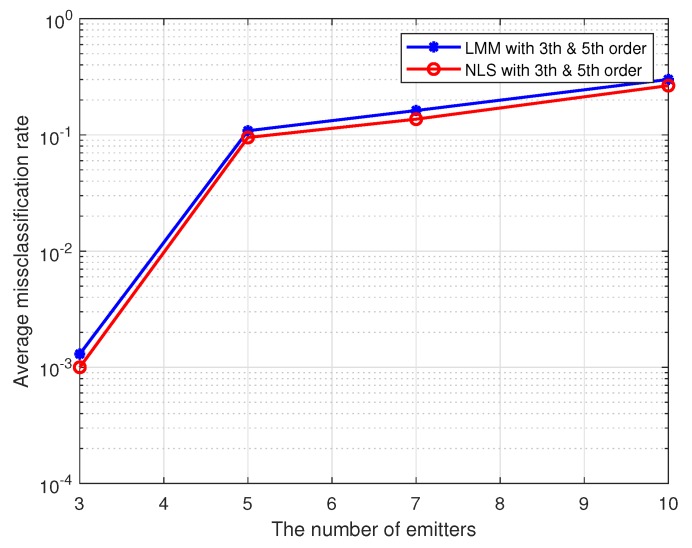
The impact of the number of emitters on the average misclassification rate for multiple transmitters.

In this simulation, we test the performance of proposed methods under the multi-user case. Here, we set the maximum order of PAs in individual emitters to 5, and 16-QAM symbols are used as the training sequence. The detailed nonlinear coefficients of each emitter are displayed in Table 1. The results are shown in Figure 8 and Figure 9, where the SNR is set to 25 dB, and each method is performed by 1000 Monte Carlo simulations.

As we can see from the Figure 8 and Figure 9, our proposed approaches are also robust and applicable to the multi-user case, and average misclassification rates of them are both increased as the number of emitters increased. Obviously, the greater the difference in nonlinear characteristics between emitters, the higher the resolution of the proposed approaches. In this simulation, as far as the nonlinear coefficients in Table 1 are concerned, the proposed approaches can resolve up to seven emitters at most.

#### 6.2.3. The Confirmation of Practical Discussions

**Figure 10:**
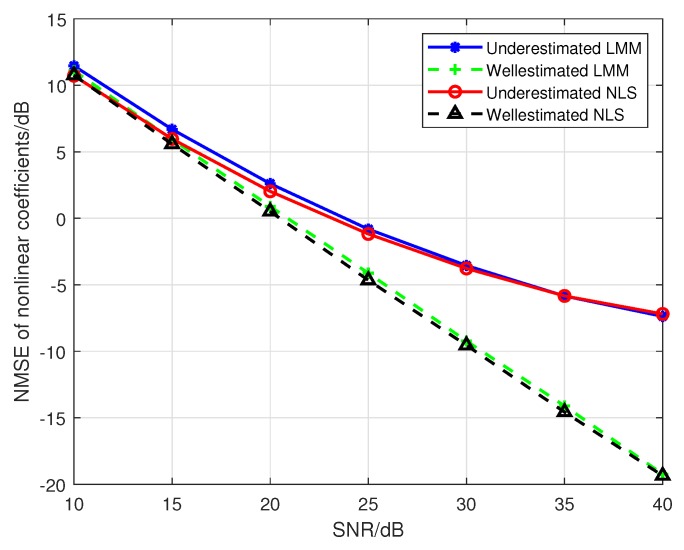
The impact of SNR on the estimation accuracy for a mismatched PA model.

**Figure 11:**
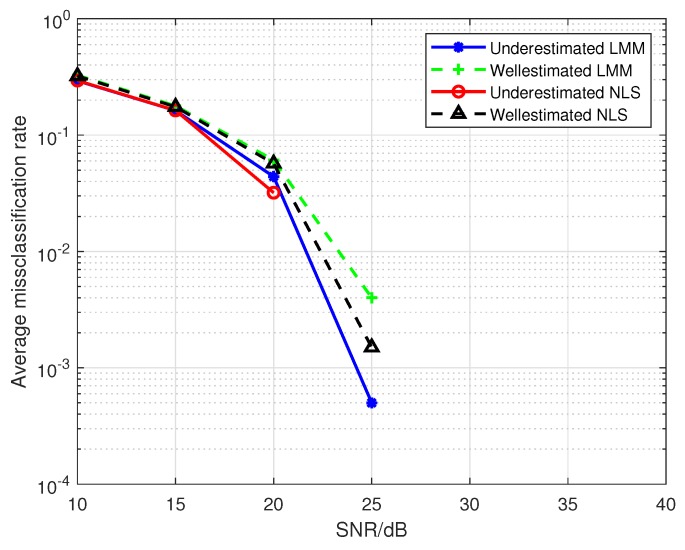
The impact of SNR on the average misclassification rate for a mismatched PA model.

Hereafter, we set the number of emitters to 2 (i.e., emitter1 and emitter2), the maximum order of the PA model *P* to 5. The first to fifth order parameters are selected from Table 1. When we use a PA model with $P_{0}=3$ to fit the nonlinearity of the emitters, the results of Figure 10 confirm the conclusion in the Section 4.2 that the underdetermined order of the PA model indeed leads to the biases in the estimations of PA parameters. However, the results in Figure 11 show that the classification performance can be improved by using the biased estimations as features, which reveals that the biases in Equation (36) can make the difference between the classification features of emitters larger. Where the training sequence is 16-QAM symbols, the estimated third-order are used as classification features, and the legends “Well-estimated LMM(NLS)” in Figure 10 and Figure 11 denote that results obtained from a well-determined order of PA model with $P_{0}=P=3$.

**Figure 12:**
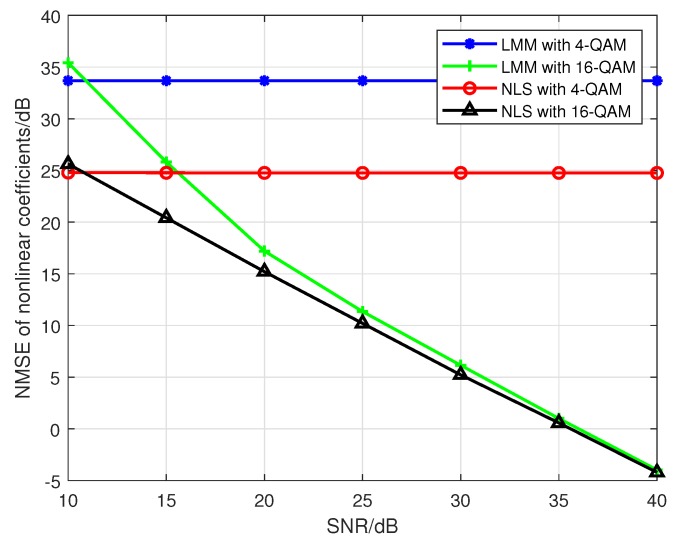
The impact of SNR on the estimation accuracy for rank-deficient approaches.

Then, according to the Proposition 2, when we use a PA model with $P_{0}=P=5$ to fit the nonlinearity of emitters, if 4-QAM, which is a constant modulus modulation, is adopted by each emitter, then it is clear that the $D_{s}$ and J(z) are both rank-deficient, and there is no unique solution in this case. Here, we compare the estimation accuracy of when taking 4-QAM and 16-QAM symbols as training sequence; the results are shown in Figure 12, which obviously corroborates the correctness of Proposition 2.

### 6.3. Experimental Results

**Figure 13:**
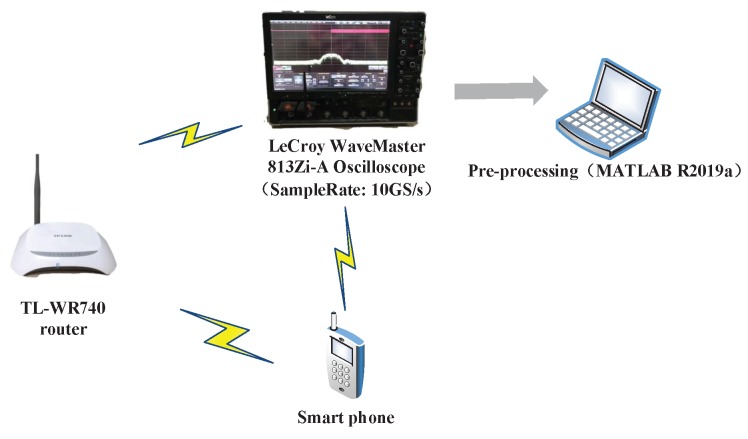
The 802.11.g-based experiment platform.

**Figure 14:**
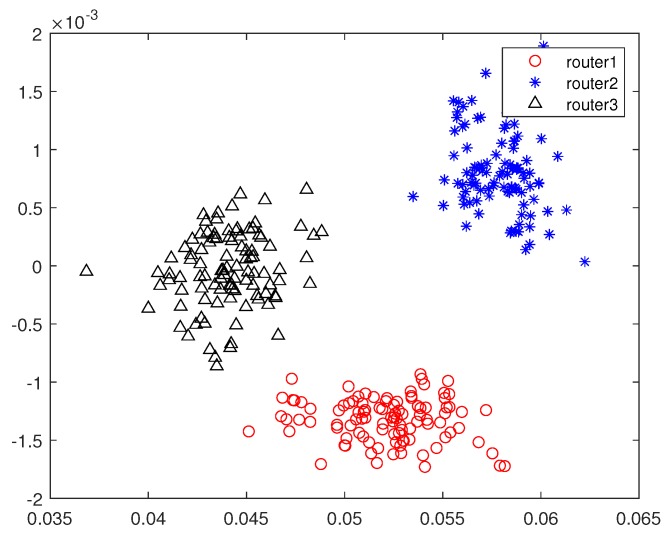
The scatter plot of the estimated 3rd order coefficients for three routers.

**Figure 15:**
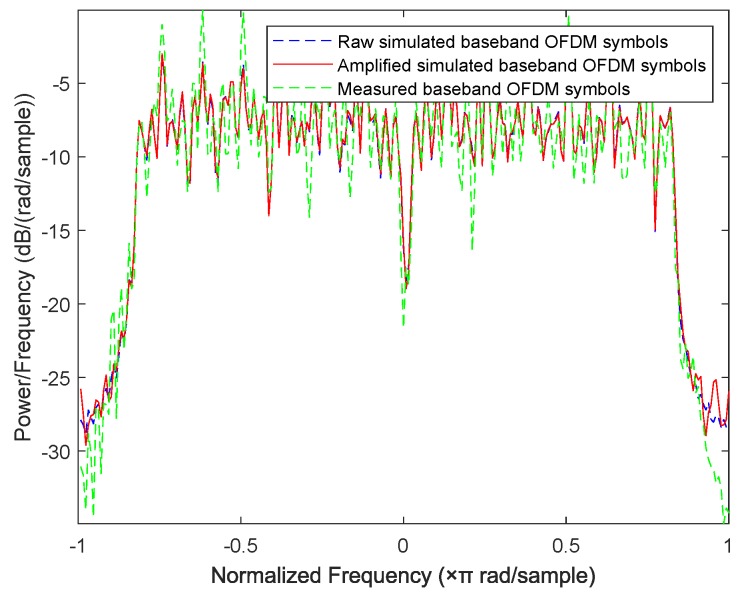
Comparison of power spectral density between measured data and simulated data for router 1.

**Figure 16:**
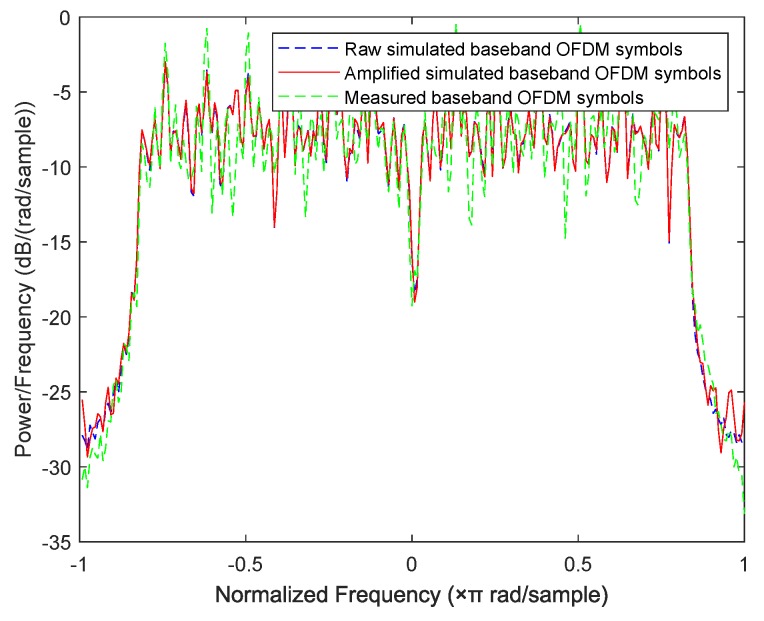
Comparison of power spectral density between measured data and simulated data for router 2.

**Figure 17:**
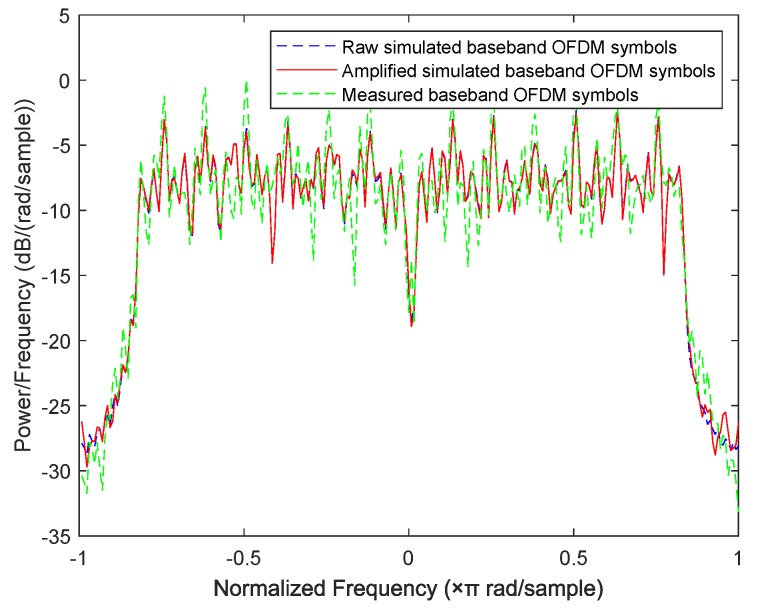
Comparison of power spectral density between measured data and simulated data for router 3.

In this subsection, we design a preliminary verification experiment to explore the effectiveness of our proposed approaches in reality. Due to the limitation of single-channel acquisition, we limit the proposed approaches in the SISO-OFDM system and validate them in a 802.11.g-based wireless local area network (WLAN) that is very common in real life. Therefore, we build an experiment platform, which is shown in Figure 13, to collect the measured router data. In this platform, we first use a LeCroy WaveMaster 813Zi-A Oscilloscope (Chestnut Ridge, NY, USA) equipped with a single antenna to acquire the RF signals from three TL-WR740 routers communicating with a smart phone on the 2.412 GHz channel, respectively. In this experiment, we use the extended NLS approach as an example to estimate nonlinear coefficients of each router, here we use the “wlan toolbox” in MATLAB R2019a (MathWorks, Natick, Massachusetts, USA) to perform pre-processing such as timing, synchronization, and de-frequency offset on the acquired RF signals. Since the nonlinearity of PA mainly caused by $IP_{3}$, we set P=3. Finally, a MEP-based classifier is used to identify the individual router. Note that the memoryless polynomial model may not be able to describe the nonlinearity of the PA in a broadband WLAN system, whereas Table 2 indicates that, according to the estimated PA coefficients in Figure 14, the mean is far greater than the variance for each router, thus the three routers are identifiable based on the extended NLS approach. Moreover, the average misclassification rates of the three routers are all 0. Therefore, we can conclude that the mismath of PA model does not affect classification performance. In addition, we also compare the power spectral density among measured data and simulated data for three routers in Figure 15, Figure 16 and Figure 17, respectively, where the legend “Measured baseband OFDM symbols” denotes downconverted acquired RF signals, the legend “Amplified simulated baseband OFDM symbols” denotes simulated baseband OFDM signals amplified by the PA with measured nonlinear coefficients, and the legend “Raw simulated baseband OFDM symbols” denotes raw simulated baseband OFDM signals, all of their power being normalized. In order to explain the results in Figure 15, Figure 16 and Figure 17, we calculate the NMSE between the PSDs of “Amplified simulated baseband OFDM symbols” and that of “Measured baseband OFDM symbols” for each router, and the NMSEs of three routers are, respectively, −7.6472, −7.5301 and −7.3589 dB, which reveal that the PSD of the signal reconstructed by using the estimated PA coefficient can well fit that of the measured signal.

## 7. Conclusions

This paper investigates the SEI scheme for multiple-antenna communication emitters, using PA nonlinearity as RFF features with the assumption that all PAs of a multiple-antenna emitter are independent from each other. Both the LMM and the NLS approaches are proposed to estimate the nonlinear coefficients in association with the memoryless polynomial PA model, where a closed-form solution can be obtained by the LMM approach, and the alternative NLS approach achieves better performance by adopting a regularized Newton–Gauss scheme. Practical discussion on the PA model mismatch is presented, and some theoretical results about the estimate bias and rank-deficient condition are provided to guide the design and implementation of the SEI over MIMO channels. In addition, an error rate analysis is also introduced for the MEP classifier. Simulation results demonstrate that the proposed approaches outperform the other existing schemes, especially in the rank-deficient case, and are effective to deal with SEI in MIMO communication systems. Moreover, the proposed approaches are verified to be effective on a 802.11.g-based experiment platform.

## Figures and Tables

**Table 1:** Nonlinear coefficients of the separate PA for each RF chain.

	$\alpha_{3,1}$	$\alpha_{3,2}$	$\alpha_{5,1}$	$\alpha_{5,2}$
Emitter1	0.0210−0.0365i	0.0290−0.0503i	−0.0099−0.0011i	−0.0158−0.0018i
Emitter2	0.0182+0.0028i	0.0131+0.0020i	0.0014−0.0008i	0.0009−0.0005i
Emitter3	0.0780−0.0488i	0.0566−0.0354i	−0.0111−0.0384i	−0.0070−0.0242i
Emitter4	0.0270−0.0247i	0.0196−0.0179i	0.0001−0.0063i	0.0000−0.0040i
Emitter5	0.0241+0.0393i	0.0174+0.0285i	0.0023−0.0097i	0.0014−0.0061i
Emitter6	0.0514−0.0520i	0.0372−0.0377i	0.0112−0.0226i	0.0070−0.0143i
Emitter7	0.0129−0.0167i	0.0102−0.0132i	−0.0006−0.0024i	−0.0004−0.0015i
Emitter8	0.0246+0.0226i	0.0195+0.0179i	0.0011−0.0062i	0.0007−0.0039i
Emitter9	0.0335+0.0410i	0.0266+0.0326i	0.0081−0.0137i	0.0051−0.0086i
Emitter10	0.0256−0.0069i	0.0204−0.0055i	0.0022−0.0033i	0.0014−0.0021i

**Table 2:** The mean and variance of the estimated nonlinear coefficients in Figure 14 for the three routers.

	Router1	Router2	Router3
Mean	0.0522−0.0013i	0.0579+0.0008i	0.0441−0.0000i
Variance	$7.1501e^{-6}$	$2.6937e^{-6}$	$3.9458e^{-6}$

## Appendix A

In this appendix, we give a simplified example for the composition of the matrix in the proposed LMM approach. Here, the parameters are assigned as R=2,J=2,L=2 and P=7. Therefore, the matrix $d_{s}$ should be as

(A1) $$d_{s}=\left[\begin{array}{cc} d_{temp} & 0_{1\times 8}\\ 0_{1\times 8} & d_{temp} \end{array}\right],$$

where $0_{1\times 8}$ is a 1×8 zero vector, $d_{temp}=\left[G_{1},G_{2}\right]$, and $G_{j}\in C^{N\times (P+1)/2}$, j=1,2, is as follows:

(A2) $$G_{j}=\left[\begin{array}{ccc} {\bar{s}}_{j}\left(0\right) & \ldots & {\left|{\bar{s}}_{j}\left(0\right)\right|}^{P-1}{\bar{s}}_{j}\left(0\right)\\ {\bar{s}}_{j}\left(1\right) & \ldots & {\left|{\bar{s}}_{j}\left(1\right)\right|}^{P-1}{\bar{s}}_{j}\left(1\right)\\ \vdots & \ddots & \vdots \\ {\bar{s}}_{j}\left(N-1\right) & \ldots & {\left|{\bar{s}}_{j}\left(N-1\right)\right|}^{P-1}{\bar{s}}_{j}\left(N-1\right) \end{array}\right].$$

Then, $D_{s}$ is

(A3) $$D_{s}=blkdiag\left(d_{s},d_{s}\right),$$

which is actually the same as $D_{s}^{\left(1,3,5,7\right)}$.

As for the case where the order of the PA order is overdetermined, note that the parameters are assigned as R=2,J=2,L=2 and P=5, whereas $P_{0}$ equals 7, then $D_{s}^{\left(1,\ldots ,P_{0}\right)}$ is equivalent to the matrix $D_{s}^{\left(1,3,5,7\right)}$ in Equation (A3). $D_{s}^{\left(1,\ldots ,P\right)}$ and $D_{s}^{\left(P+2,\ldots ,P_{0}\right)}$ are respectively written as

(A4a)Ds(1,3,5)=blkdiagds(1,3,5),ds(1,3,5),(A4b)Ds(7)=blkdiagds(7),ds(7),

where

(A5a)ds(1,3,5)=e10(1),e10(2),e01(1),e01(2),(A5b)ds(7)=f10(1),f10(2),f01(1),f01(2),

and $e_{10}^{\left(j\right)}$, $e_{01}^{\left(j\right)}$, $f_{10}^{\left(j\right)}$, and $f_{01}^{\left(j\right)}$(j=1,2) are respectively expressed by:

(A6) $$e_{10}^{\left(j\right)}=\left[\begin{array}{ccc} {\bar{s}}_{j}\left(0\right) & \ldots & {\left|{\bar{s}}_{j}\left(0\right)\right|}^{4}{\bar{s}}_{j}\left(0\right)\\ {\bar{s}}_{j}\left(1\right) & \ldots & {\left|{\bar{s}}_{j}\left(1\right)\right|}^{4}{\bar{s}}_{j}\left(1\right)\\ \vdots & \ddots & \vdots \\ {\bar{s}}_{j}\left(N-1\right) & \ldots & {\left|{\bar{s}}_{j}\left(N-1\right)\right|}^{4}{\bar{s}}_{j}\left(N-1\right)\\ 0 & \ldots & 0 \end{array}\right],$$

(A7) $$e_{01}^{\left(j\right)}=\left[\begin{array}{ccc} 0 & \ldots & 0\\ {\bar{s}}_{j}\left(0\right) & \ldots & {\left|{\bar{s}}_{j}\left(0\right)\right|}^{4}{\bar{s}}_{j}\left(0\right)\\ {\bar{s}}_{j}\left(1\right) & \ldots & {\left|{\bar{s}}_{j}\left(1\right)\right|}^{4}{\bar{s}}_{j}\left(1\right)\\ \vdots & \ddots & \vdots \\ {\bar{s}}_{j}\left(N-1\right) & \ldots & {\left|{\bar{s}}_{j}\left(N-1\right)\right|}^{4}{\bar{s}}_{j}\left(N-1\right) \end{array}\right],$$

(A8) $$f_{10}^{\left(j\right)}=\left[\begin{array}{c} {\left|{\bar{s}}_{j}\left(0\right)\right|}^{6}{\bar{s}}_{j}\left(0\right)\\ {\left|{\bar{s}}_{j}\left(1\right)\right|}^{6}{\bar{s}}_{j}\left(1\right)\\ \vdots \\ {\left|{\bar{s}}_{j}\left(N-1\right)\right|}^{6}{\bar{s}}_{j}\left(N-1\right)\\ 0 \end{array}\right]$$

(A9) $$f_{01}^{\left(j\right)}=\left[\begin{array}{c} 0\\ {\left|{\bar{s}}_{j}\left(0\right)\right|}^{6}{\bar{s}}_{j}\left(0\right)\\ {\left|{\bar{s}}_{j}\left(1\right)\right|}^{6}{\bar{s}}_{j}\left(1\right)\\ \vdots \\ {\left|{\bar{s}}_{j}\left(N-1\right)\right|}^{6}{\bar{s}}_{j}\left(N-1\right) \end{array}\right].$$

Similarly, as for the case that the order of the PA model is underdetermined, be aware that the parameters are assigned as R=2,J=2,L=2 and P=7, whereas $P_{0}$ equals 5, then $D_{s}^{\left(1,\ldots ,P\right)}$ is equivalent to the matrix $D_{s}^{\left(1,3,5,7\right)}$ in Equation (A3). $D_{s}^{\left(1,\ldots ,P_{0}\right)}$ and $D_{s}^{\left(P_{0}+2,\ldots ,P\right)}$ are equivalent to the matrix $D_{s}^{\left(1,3,5\right)}$ and $D_{s}^{\left(7\right)}$ in Equation (A4a) and Equation (A4b), respectively.

The examples for $h_{\alpha }^{\left(1,3,5\right)}$ and $h_{\alpha }^{\left(7\right)}$ are shown as

(A10a)hα(1,3,5)=w1,0(3)αvec(1,3,5)T,w1,1(3)αvec(1,3,5)T,w2,0(3)αvec(1,3,5)T,w2,1(3)αvec(1,3,5)TT(A10b)hα(7)=w1,0(1)αvec(7)T,w1,1(1)αvec(7)T,w2,0(1)αvec(7)T,w2,1(1)αvec(7)TT

and

(A11a)wr,lh(1)=diaghr1(lh),hr2(lh)(A11b)wr,lh(3)=blkdiaghr1(lh)I3,hr2(lh)I3(A11c)αvec(1,3,5)=1,α3,1,α5,1,1,α3,2,α5,2T(A11d)αvec(7)=α7,1,α7,2T,

where diag(·) denotes a diagonal function, $I_{3}$ is a 3×3 unit matrix, r=1,2 and $l_{h}=0,1$.

## Appendix B

In this appendix, we first give the basis of Guess 1.

**Proof.** At first, we discuss the case where the parameters are set to be J=1,L=2 and P=7, respectively. As mentioned in Appendix A, $d_{s}^{\left(1,3,5\right)}$ and $d_{s}^{\left(7\right)}$ can be expressed as block matrices as follows:

(A12a)ds(1,3,5)=e10(1),e01(1),(A12b)ds(7)=f10(1),f01(1).

We thus have

(A13) $$\begin{array}{c} {\left(d_{s}^{\left(1,3,5\right)}\right)}^{†}d_{s}^{\left(7\right)}\\ ={\left(\left[\begin{array}{c} {\left(e_{10}^{\left(1\right)}\right)}^{H}\\ {\left(e_{01}^{\left(1\right)}\right)}^{H} \end{array}\right]\left[e_{10}^{\left(1\right)},e_{01}^{\left(1\right)}\right]\right)}^{-1}\left[\begin{array}{c} {\left(e_{10}^{\left(1\right)}\right)}^{H}\\ {\left(e_{01}^{\left(1\right)}\right)}^{H} \end{array}\right]\left[f_{10}^{\left(1\right)},f_{01}^{\left(1\right)}\right]\\ =\;\;\;\;\;\;{\left[\begin{array}{cc} A & V\\ V^{H} & A \end{array}\right]}^{-1}\left[\begin{array}{cc} E & F_{1}\\ F_{2} & E \end{array}\right], \end{array}$$

where

(A14a)A=e10(1)He10(1)=e01(1)He01(1),(A14b)V=e10(1)He01(1),(A14c)E=e10(1)Hf10(1)=e01(1)Hf01(1),(A14d)F1=e10(1)Hf01(1),(A14e)F2=e01(1)Hf10(1),

with ${\left(\cdot \right)}^{-1}$ being the inverse of a matrix. Then, according to the mathematic formula for the inverse of the block matrix, we get

(A15) $${\left[\begin{array}{cc} A & V\\ V^{H} & A \end{array}\right]}^{-1}=\left[\begin{array}{cc} \Pi_{1} & -\Pi_{1}VA^{-1}\\ -\Pi_{2}V^{H}A^{-1} & \Pi_{2} \end{array}\right],$$

with

(A16) $$\Pi_{1}={\left(A-VA^{-1}V^{H}\right)}^{-1},$$

(A17) $$\Pi_{2}={\left(A-V^{H}A^{-1}V\right)}^{-1}.$$

Therefore, if we guess that

(A18a)F1=VA−1E,(A18b)F2=VHA−1E,

which is actually valid for 16-QAM symbols in practice, then the matrix ${\left(d_{s}^{\left(1,3,5\right)}\right)}^{†}d_{s}^{\left(7\right)}$ is a block diagonal matrix. As a result, for arbitrary *J* and *L*, we can guess that

(A19) $${\left(D_{s}^{\left(1,\ldots ,P_{0}\right)}\right)}^{†}D_{s}^{\left(P_{0}+2,\ldots ,P\right)}=blkdiag\left(\underset{RJL}{\underset{︸}{c,\ldots ,c,c}}\right).$$

 □

Next, we give the proof of Proposition 1.

**Proof.** When the order of the PA model is underdetermined (i.e., $P_{0}<P$), according to Equation (24), ignoring the white noise term, if the Guess 1 holds true, then the *j*th submatrix of Q in Equation (13) can be expanded into

(A20) $$\begin{array}{c} \;Q_{j}\\ ={Q_{j}}^{\left(1,\ldots ,P_{0}\right)}+{Q_{j}}^{\left(P_{0}+2,\ldots ,P\right)}\\ =\left[h_{1j}\left(0\right)\left(\alpha_{j}^{\left(1,\ldots ,P_{0}\right)}+\lambda_{j}\right),\ldots ,h_{Rj}\left(L-1\right)\left(\alpha_{j}^{\left(1,\ldots ,P_{0}\right)}+\lambda_{j}\right)\right], \end{array}$$

where ${Q_{j}}^{\left(a,\ldots ,b\right)}$ and ${\alpha_{j}}^{\left(a,\ldots ,b\right)}$ denote the $Q_{j}$ and $\alpha_{j}$ that are populated by the *a*th to the *b*th order PA parameters, respectively. $\lambda_{j}\in C^{m}$ is populated by the $\left(P_{0}+2\right)$th to the *P*th order PA parameters and the matrix c in Equation (A19), whose *i*th (i=1,2,…,m) element is as $\lambda_{j}\left(i\right)=c_{i1}\alpha_{P_{0}+2,j}+\ldots +c_{in}\alpha_{P,j}$. Since the $Q_{j}$ is a matrix with rank 1, the first left singular vector $U_{j}^{\left(:,1\right)}$ of $Q_{j}$ obtained by SVD is equivalent to the eigenvector corresponding to the non-zero eigenvalue of the matrix $Q_{j}{Q_{j}}^{H}$. Thus, the $U_{j}^{\left(:,1\right)}$ of Equation (A20) can be computed by the eigenvalue decomposition (EVD) of $Q_{j}{Q_{j}}^{H}$, which is as

(A21) $$U_{j}^{\left(:,1\right)}={\left[1+\lambda_{j}\left(1\right),\alpha_{3,j}+\lambda_{j}\left(2\right),\ldots ,\alpha_{P_{0},j}+\lambda_{j}\left(m\right)\right]}^{T},$$

then, according to Equation (14), we have biased ${\hat{\alpha }}_{j}$ as

(A22) $${\hat{\alpha }}_{j}={\left[1,\frac{\alpha_{3,j}+\lambda_{j}\left(2\right)}{1+\lambda_{j}\left(1\right)},\ldots ,\frac{\alpha_{P_{0},j}+\lambda_{j}\left(m\right)}{1+\lambda_{j}\left(1\right)}\right]}^{T}.$$

Eventually, we can obtain the biases as shown in Equations (26a)–(26c). □

## Appendix C

In this appendix, we give the proof for Proposition 2.

At first, we prove that, when the training sequence, consisting of QAM symbols, has *M* different modulus values and the maximum order of the PA model is *P*, if M<(P+1)/2, then the matrix $D_{s}$ is rank deficient.

**Proof.** At first, we consider a matrix $B\in C^{l_{B}\times c_{B}}$, which is

(A23) $$B=\left[\begin{array}{ccccc} 1 & b_{1} & b_{1}^{2} & \ldots & b_{1}^{c_{B}-1}\\ 1 & b_{2} & b_{2}^{2} & \ldots & b_{2}^{c_{B}-1}\\ \vdots & \vdots & \vdots & \ddots & \vdots \\ 1 & b_{l_{B}} & b_{l_{B}}^{2} & \ldots & b_{l_{B}}^{c_{B}-1} \end{array}\right].$$

when $l_{B}=c_{B}$, B is actually a Vandermonde matrix and its determinant can be expressed as

(A24) $$det\left(B\right)=\prod_{1\leq k<i\leq c_{B}}\left(b_{i}-b_{k}\right).$$

Therefore, if the level of the amplitude of $b_{i}$$\left(i=1,\ldots ,l_{B}\right)$ is less than $c_{B}$, then det(B) equals 0 and B is definitely singular. When $l_{B}>c_{B}$, if the level of the amplitude of $b_{i}$$\left(i=1,\ldots ,l_{B}\right)$ is less than $c_{B}$, then any $c_{B}\times c_{B}$ sub-matrix of B is a singular Vandermonde matrix, that is, any $c_{B}\times c_{B}$ sub-matrix of B is column linear correlation, then B is also column linear correlation, which means B is a column rank-deficient matrix. Now, we consider the matrix $G_{j}\in^{N\times (P+1)/2}$ in Equation (A2), here j=1,…,J, and it can be rewritten by

(A25) $$\begin{array}{cc} G_{j} & =G_{j}^{\left(1\right)}G_{j}^{\left(2\right)}\;\\ & =G_{j}^{\left(1\right)}\left[\begin{array}{cccc} 1 & {\left|{\bar{s}}_{j}\left(0\right)\right|}^{2} & \ldots & {\left|{\bar{s}}_{j}\left(0\right)\right|}^{P-1}\\ 1 & {\left|{\bar{s}}_{j}\left(1\right)\right|}^{2} & \ldots & {\left|{\bar{s}}_{j}\left(1\right)\right|}^{P-1}\\ \vdots & \vdots & \ddots & \vdots \\ 1 & {\left|{\bar{s}}_{j}\left(N-1\right)\right|}^{2} & \ldots & {\left|{\bar{s}}_{j}\left(N-1\right)\right|}^{P-1} \end{array}\right] \end{array},$$

where $G_{j}^{\left(1\right)}=diag\left({\bar{s}}_{j}\left(0\right),{\bar{s}}_{j}\left(1\right),\ldots ,{\bar{s}}_{j}\left(N-1\right)\right)$ is a reversible diagonal matrix. In addition, $G_{j}^{\left(2\right)}$ is equivalent to the matrix B when we set $b_{i}={\left|{\bar{s}}_{j}\left(i-1\right)\right|}^{2}$, i=1,2,…,N. Since the training sequence is composed of QAM symbols that have only *M* different moduli values, then any $\frac{P+1}{2}\times \frac{P+1}{2}$ sub-matrix of $G_{j}^{\left(2\right)}$ is a singular Vandermonde matrix when M<(P+1)/2. Thus, $G_{j}$ is column rank-deficient, and, according to Appendix A, $d_{temp}=\left[G_{1},G_{2},\ldots ,G_{J}\right]$ is also column rank-deficient. Eventually, $d_{s}$ and $D_{s}$ are both column rank-deficient due to the linear correlation among their column vectors. However, when M≥(P+1)/2, as long as *N* is large enough, then at least one of $\frac{P+1}{2}\times \frac{P+1}{2}$ sub-matrix of the $G_{j}^{\left(2\right)}$ is a non-singular Vandermonde matrix. Thus, the $G_{j}^{\left(2\right)}$ is a column full-rank matrix, and $D_{s}$ is also a column full-rank matrix. □

Next, we prove that when the training sequence, QAM symbols, has *M* different modulus values and the maximum order of the PA model is *P*, if M<(P+1)/2, then the matrix J(z) is rank-deficient at every z.

**Proof.** When the number of transmit antennas is *J*, then the Jacobian matrix J(z) can be computed from g(z) in Equation (15) as

(A26) $$J\left(z\right)=\left[\begin{array}{ccccc} \kappa & 0 & \ldots & 0 & \chi_{1}\\ 0 & \kappa & \vdots & \vdots & \chi_{2}\\ \vdots & \vdots & \ddots & 0 & \vdots \\ 0 & \ldots & 0 & \kappa & \chi_{R} \end{array}\right]$$

with

(A27) $$\kappa =\left[O^{\left(0\right)},\ldots ,O^{\left(L-1\right)}\right],$$

(A28) $$\chi_{r}=\left[{W_{r}}^{\left(0\right)}+\cdots +{W_{r}}^{\left(L-1\right)}\right],$$

where $O^{\left(l_{h}\right)}$ and ${W_{r}}^{\left(l_{h}\right)}$$\left(l_{h}=0,1,\ldots ,L-1\right)$ are respectively shown as

(A29) $$\begin{array}{c} O^{\left(l_{h}\right)}=\left[O_{1}^{\left(l_{h}\right)},O_{2}^{\left(l_{h}\right)},\ldots ,O_{J}^{\left(l_{h}\right)}\right], \end{array}$$

(A30) $${W_{r}}^{\left(l_{h}\right)}=\left[W_{r1}^{\left(l_{h}\right)},W_{r2}^{\left(l_{h}\right)},\ldots ,W_{rJ}^{\left(l_{h}\right)}\right].$$

Note that $O_{j}^{\left(l_{h}\right)}$ and $W_{rj}^{\left(l_{h}\right)}$$\left(l_{h}=0,1,\ldots ,L-1\right)$ are

(A31) $$\begin{array}{c} O_{j}^{\left(l_{h}\right)}=\Lambda_{l_{h}}{\left[\underset{l_{h}}{\underset{︸}{0,\ldots ,0}},\varphi \left({\bar{s}}_{j}\left(0\right)\right),\ldots ,\varphi \left({\bar{s}}_{j}\left(N-1\right)\right),\underset{L-1-l_{h}}{\underset{︸}{0,\ldots ,0}}\right]}^{T}, \end{array}$$

(A32) $$W_{rj}^{\left(l_{h}\right)}=h_{rj}\left(l_{h}\right)\Lambda_{l_{h}}\left[\begin{array}{c} 0_{l_{h}\times \left(P-1\right)/2}\\ \psi_{s}\\ 0_{\left(L-1-l_{h}\right)\times \left(P-1\right)/2} \end{array}\right],$$

respectively, and

(A33) $$\begin{array}{c} \varphi \left({\bar{s}}_{j}\left(n\right)\right)=1+\alpha_{3,j}{\left|{\bar{s}}_{j}\left(n\right)\right|}^{2}+\alpha_{5,j}{\left|{\bar{s}}_{j}\left(n\right)\right|}^{4}+\cdots +\alpha_{P,j}{\left|{\bar{s}}_{j}\left(n\right)\right|}^{P-1}, \end{array}$$

(A34) $$\Lambda_{l_{h}}=diag\left(\underset{l_{h}}{\underset{︸}{1,\ldots ,1}},{\bar{s}}_{j}\left(0\right),\ldots ,{\bar{s}}_{j}\left(N-1\right),\underset{L-1-l_{h}}{\underset{︸}{1,\ldots ,1}}\right),$$

(A35) $$\begin{array}{c} \psi_{s}=\left[\begin{array}{cccc} {\left|{\bar{s}}_{j}\left(0\right)\right|}^{2} & {\left|{\bar{s}}_{j}\left(0\right)\right|}^{4} & \ldots & {\left|{\bar{s}}_{j}\left(0\right)\right|}^{P-1}\\ {\left|{\bar{s}}_{j}\left(1\right)\right|}^{2} & {\left|{\bar{s}}_{j}\left(1\right)\right|}^{4} & \ldots & {\left|{\bar{s}}_{j}\left(1\right)\right|}^{P-1}\\ \vdots & \vdots & \ddots & \vdots \\ {\left|{\bar{s}}_{j}\left(N-1\right)\right|}^{2} & {\left|{\bar{s}}_{j}\left(N-1\right)\right|}^{4} & \ldots & {\left|{\bar{s}}_{j}\left(N-1\right)\right|}^{P-1} \end{array}\right]. \end{array}$$

If we define a matrix $J_{r}$ as

(A36) $$J_{r}=\left[O^{\left(0\right)},\ldots ,O^{\left(L-1\right)},{W_{r}}^{\left(0\right)}+\cdots +{W_{r}}^{\left(L-1\right)}\right],$$

then, as for $\Theta_{j}$, a sub-matrix of $J_{r}$, composed of the elements corresponding to the *j*th transmit antenna, which is

(A37) $$\Theta_{j}=\left[O_{j}^{\left(0\right)},O_{j}^{\left(1\right)},\ldots ,O_{j}^{\left(L-1\right)},W_{rj}^{\left(0\right)}+W_{rj}^{\left(1\right)}+\cdots +W_{rj}^{\left(L-1\right)}\right].$$

Similar to the discussion of the matrix B in Equation (A23), as for the matrix Ω,

(A38) $$\Omega =\left[\begin{array}{cccc} \varphi \left({\bar{s}}_{j}\left(0\right)\right) & h_{1j}\left(l_{h}\right){\left|{\bar{s}}_{j}\left(0\right)\right|}^{2} & \ldots & h_{1j}\left(l_{h}\right){\left|{\bar{s}}_{j}\left(0\right)\right|}^{P-1}\\ \varphi \left({\bar{s}}_{j}\left(1\right)\right) & h_{1j}\left(l_{h}\right){\left|{\bar{s}}_{j}\left(1\right)\right|}^{2} & \ldots & h_{1j}\left(l_{h}\right){\left|{\bar{s}}_{j}\left(1\right)\right|}^{P-1}\\ \vdots & \vdots & \ddots & \vdots \\ \varphi \left({\bar{s}}_{j}\left(N-1\right)\right) & h_{1j}\left(l_{h}\right){\left|{\bar{s}}_{j}\left(N-1\right)\right|}^{2} & \ldots & h_{1j}\left(l_{h}\right){\left|{\bar{s}}_{j}\left(N-1\right)\right|}^{P-1} \end{array}\right]$$

when the training sequence is composed of QAM symbols that have only *M* different moduli values and N≥(P+1)/2, if M<(P+1)/2, then there are at least (P+1)/2−M+1 identical row vectors in any $\frac{P+1}{2}\times \frac{P+1}{2}$ sub-matrix of Ω, which means that any $\frac{P+1}{2}\times \frac{P+1}{2}$ sub-matrix of Ω is a singular matrix, and the column vectors of it are also considered to be linear correlation. As a result, Ω is also column linear correlation, that is, the Ω is a column rank-deficient matrix. Since the matrix Ω has been proved to be a column rank-deficient matrix, the matrix $\left[O_{j}^{\left(l_{h}\right)},W_{rj}^{\left(l_{h}\right)}\right]$ is obviously column rank-deficient, and there must be a set of coefficients $\left\{\eta_{1},\eta_{2},\ldots ,\eta_{(P+1)/2}\right\}$ that are not all equal to 0. Consequently, the following formula holds

(A39) $$\eta_{1}O_{j}^{\left(l_{h}\right)}+\sum_{c_{W}=1}^{(P-1)/2}\eta_{c_{W}+1}W_{rj}^{\left(l_{h}\right)}\left(:,c_{W}\right)=0,$$

where $W_{rj}^{\left(l_{h}\right)}\left(:,c_{W}\right)$ denotes the $c_{W}$-th column of the matrix $W_{rj}^{\left(l_{h}\right)}$. Therefore, for the matrix $\Theta_{j}$, there are also a set of coefficients $\left\{\underset{L}{\underset{︸}{\eta_{1},\eta_{1},\ldots ,\eta_{1}}},\eta_{2},\ldots ,\eta_{(P+1)/2}\right\}$ that are not all equal to 0 to make the following expression valid, i.e.,

(A40) $$\eta_{1}\sum_{l_{h}=0}^{L-1}O_{j}^{\left(l_{h}\right)}+\sum_{c_{W}=1}^{(P-1)/2}\eta_{c_{W}+1}\Gamma_{c_{W}}=0$$

with

(A41) $$\Gamma_{c_{W}}=\sum_{l_{h}=0}^{L-1}W_{rj}^{\left(l_{h}\right)}\left(:,c_{W}\right).$$

Then, we can conclude that the matrix $\Theta_{j}$ is rank-deficient and the matrix $\left[\Theta_{1},\Theta_{2},\ldots ,\Theta_{J}\right]$ is also rank-deficient. Thus, the Jacobian matrix $J_{r}$ is also rank-deficient because $J_{r}$ is the elementary column transformation of the matrix $\left[\Theta_{1},\Theta_{2},\ldots ,\Theta_{J}\right]$ and the elementary column transformation does not change the rank of the matrix. Eventually, the the Jacobian matrix J(z) in Equation (A26) is rank-deficient. □
